# Exploring direct and indirect targets of current antileishmanial drugs using a novel thermal proteomics profiling approach

**DOI:** 10.3389/fcimb.2022.954144

**Published:** 2022-08-03

**Authors:** Ana Victoria Ibarra-Meneses, Audrey Corbeil, Victoria Wagner, Francis Beaudry, Rubens L. do Monte-Neto, Christopher Fernandez-Prada

**Affiliations:** ^1^ Département de Pathologie et Microbiologie, Faculté de Médecine Vétérinaire, Université de Montréal, Saint-Hyacinthe, QC, Canada; ^2^ The Research Group on Infectious Diseases in Production Animals (GREMIP), Faculté de Médecine Vétérinaire, Université de Montréal, Saint-Hyacinthe, QC, Canada; ^3^ Département de Biomédecine, Faculté de Médecine Vétérinaire, Université de Montréal, Saint-Hyacinthe, QC, Canada; ^4^ Centre de recherche sur le cerveau et l’apprentissage (CIRCA), Université de Montréal, Montréal, QC, Canada; ^5^ Biotechnology Applied to Pathogens (BAP) - Instituto René Rachou – Fundação Oswaldo Cruz/Fiocruz Minas, Belo Horizonte, Minas Gerais, Brazil

**Keywords:** *Leishmania*, antileishmanial drugs, mode of action, thermal proteome profiling, drug resistance, antimony, miltefosine, amphotericin B

## Abstract

Visceral leishmaniasis (VL), caused by *Leishmania infantum*, is an oft-fatal neglected tropical disease. In the absence of an effective vaccine, the control of leishmaniasis relies exclusively on chemotherapy. Due to the lack of established molecular/genetic markers denoting parasite resistance, clinical treatment failure is often used as an indicator. Antimony-based drugs have been the standard antileishmanial treatment for more than seven decades, leading to major drug resistance in certain regions. Likewise, drug resistance to miltefosine and amphotericin B continues to spread at alarming rates. In consequence, innovative approaches are needed to accelerate the identification of antimicrobial drug targets and resistance mechanisms. To this end, we have implemented a novel approach based on thermal proteome profiling (TPP) to further characterize the mode of action of antileishmanials antimony, miltefosine and amphotericin B, as well as to better understand the mechanisms of drug resistance deployed by *Leishmania*. Proteins become more resistant to heat-induced denaturation when complexed with a ligand. In this way, we used multiplexed quantitative mass spectrometry-based proteomics to monitor the melting profile of thousands of expressed soluble proteins in WT, antimony-resistant, miltefosine-resistant, and amphotericin B-resistant *L. infantum* parasites, in the presence (or absence) of the above-mentioned drugs. Bioinformatics analyses were performed, including data normalization, melting profile fitting, and identification of proteins that underwent changes (fold change > 4) caused by complexation with a drug. With this unique approach, we were able to narrow down the regions of the *L. infantum* proteome that interact with antimony, miltefosine, and amphotericin B; validating previously-identified and unveiling novel drug targets. Moreover, analyses revealed candidate proteins potentially involved in drug resistance. Interestingly, we detected thermal proximity coaggregation for several proteins belonging to the same metabolic pathway (i.e., tryparedoxin peroxidase and aspartate aminotransferase in proteins exposed to antimony), highlighting the importance of these pathways. Collectively, our results could serve as a jumping-off point for the future development of innovative diagnostic tools for the detection and evaluation of antimicrobial-resistant *Leishmania* populations, as well as open the door for new on-target therapies.

## Introduction

Visceral leishmaniasis (VL), caused by *Leishmania infantum*, is part of the group of neglected tropical diseases (NTDs). This manifestation of leishmaniasis is fatal if left untreated. VL cases occur in 78 countries, mainly affecting (87% of global VL cases) Brazil, Eritrea, Ethiopia, India, Kenya, Somalia, South Sudan, and Sudan (WHO, 2020). In the absence of an effective vaccine, control of leishmaniases is exclusively dependent on chemotherapy. Amphotericin B (AmB), miltefosine (MF), and antimony-derivatives (Sb) are the three main drugs that have been used in the treatment of VL (Chakravarty and Sundar, 2019). However, VL is still treated primarily with antimonial drugs sodium stibogluconate and meglumine antimoniate, which were introduced as far back as the 1940s (Uliana et al., 2018), but are failing due to rapid emergence of parasite resistance (Sundar et al., 2000). Since 2013, AmB, in its liposomal formulation, has been recommended as a first-line antileishmanial drug in VL endemic areas (Van Griensven and Diro, 2019). However, its relapse rate ranges from 2.4% to 89% depending on the study in question, and in some regions, it is therefore recommended that AmB be followed by a 7-day administration of MF (Olliaro et al., 2005; Sundar and Chakravarty, 2010; Burza et al., 2014). MF is the first effective oral drug for treatment of VL. Its formulation has facilitated treatment in remote areas, though its cure rate varies (90-95%) and its relapse rate (6.8-20%) has been increasing over time (Sundar and Olliaro, 2007; Sundar et al., 2012; Rijal et al., 2013). Unfortunately, even the oft-recommended combination therapy is at risk of failure, further emphasizing the severity of drug resistance as an obstacle to treatment of leishmaniasis (Fernandez-Prada et al., 2016).

The mechanisms of drug resistance (MoDR) and mode of action (MoA) of the drugs used in treatment of VL have been extensively studied but remain unclear (Ponte-Sucre et al., 2017). Resistance to Sb-based drugs is the most well-investigated; these treatments are based upon the reduction of pro-drug Sb^V^ to its trivalent form, Sb^III^, inside macrophages and/or parasites (Haldar et al., 2011). Decreases in the expression of aquaglyceroporin AQP1 and amplification of the gene coding for ABC-transporter MRPA have both been detected in Sb-resistant parasites, leading to decreased uptake and increased sequestration of Sb^III^, respectively (Leprohon et al., 2009; Brotherton et al., 2013; Monte-Neto et al., 2015; Douanne et al., 2020b; Potvin et al., 2021). On the other hand, AmB is thought to generate pores in the lipid bilayer of parasites through preferential binding to ergosterol, leading to cell death, while MF is hypothesized to target parasite lipid metabolism (Vincent et al., 2014; Fernandez-Prada et al., 2016). That said, in the absence of reliable molecular or genetic markers related to parasite resistance, clinical treatment failure is generally used as an indicator of drug resistance (Sundar et al., 2000). Identifying these biomarkers while understanding the MoA and the MoDR would undoubtedly contribute to designing new public health strategies to prevent further development of drug resistance and facilitate the discovery (or repositioning) of more effective antileishmanial drugs (Leprohon et al., 2015). Considering that *Leishmania* gene expression is largely regulated at the post-transcriptional level, protein analysis is a powerful omic tool for understanding all the effectors potentially involved in various steps of MoA and MoDR (Fernandez-Prada et al., 2018b).

Thermal Proteomic Profiling (TPP) is a new approach with applications in drug discovery, as well as in the mapping of metabolic pathways and the study of protein-protein interactions (Mateus et al., 2020). TPP is based on the principle that proteins become more resistant to heat-induced denaturation when complexed with a ligand (i.e., drugs, nucleic acids, or proteins). The thermal change of these proteins is quantified by mass spectrometry through detection of changes in the abundance of soluble proteins (Mateus et al., 2016; Perrin et al., 2020). Taking advantage of this powerful approach, we have implemented a novel, multiplexed, quantitative mass spectrometry-based proteomics protocol to identify novel direct and off-target players potentially involved in the MoA and/or the MoDR of antileishmanial drugs. Owing to our TPP protocol (performed over the temperature range (TPP-TR) of 37–70°C, at a fixed 100 µM drug concentration) we were able to monitor the melting profile of thousands of expressed soluble proteins in drug-sensitive, Sb-, MF- and AmB-resistant *L. infantum* parasites, in the presence (or absence) of the antileishmanial drug in question. This protocol was validated by the identification of previously known drug targets and proved very useful to disclose novel players involved in the MoA/MoDR of current antileishmanial treatments.

## Material and methods

### *Leishmania* cell lines and parasite culture

**Figure 1:**
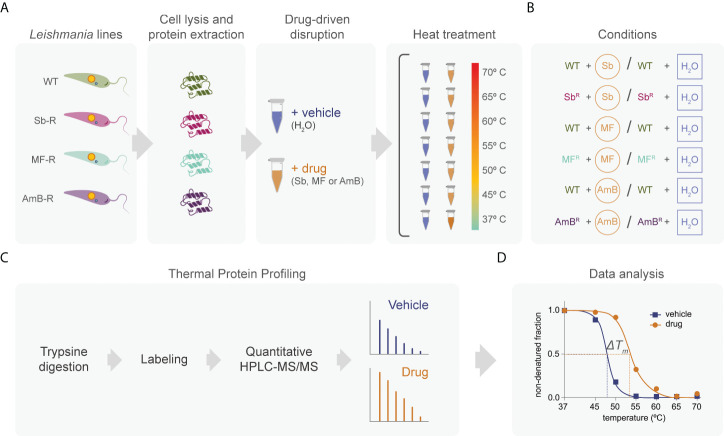
Quantitative proteome-wide profiling of leishmanial protein thermal stability in the presence or absence of anti-leishmanial drugs. Whole-parasite proteins were extracted in triplicate from *L. infantum* sensitive (WT) and resistant strains (Sb2000.1, MF200.5, and AmB1000.1), and the extracts were treated with a drug or its vehicle **(A)**. Our protocol included six different conditions including sensitive and drug-resistant parasites, in the presence or absence of drug **(B)**. For each condition, the parasite-extract sample was divided into seven aliquots that were subjected to the indicated temperatures. After digestion with trypsin, each sample was labeled using a dimethyl labeling strategy. Samples were analyzed by means of HPLC-MS/MS using a hybrid Quadrupole-Orbitrap mass spectrometer **(C)**. The resulting reporter ion intensities were used to generate melting curves and estimate the melting temperature (*T_m_*) of each individual leishmanial protein independently for each condition **(D)**.

Four different *Leishmania infantum* lines were used in this study, including: *L. infantum* wild-type (WT) strain (MHOM/MA/67/ITMAP-263) and the *in vitro* generated resistant mutants Sb2000.1 (resistant to 2000 µM Sb), MF200.5 (resistant to 200 µM MF), and AmB1000.1 (resistant to 1 µM AmB) (Leprohon et al., 2009; Brotherton et al., 2013; Brotherton et al., 2014; Fernandez-Prada et al., 2016). Briefly, *Leishmania* parasites were grown at 25°C in M199 medium supplemented with 10% fetal bovine serum (Wisent) and 5 μg/mL hemin (Millipore) at pH 7.0 until mid-log density (2.5 ×10^9^ parasites). Drug-resistant strains were grown in the presence of the drug for which they had been previously selected: for Sb2000.1, 2 mM Sb (potassium antimonyl tartrate, Sigma-Aldrich #383376) was added; for MF200.5, 200 μM MF (miltefosine, Cayman Chem #63280); for AmB1000.1, 1 µM AmB (amphotericin B solution, Sigma #A2942). Once the logarithmic phase was reached, each culture was divided into two 500 mL aliquots. Cultures were then submitted to multiple centrifugation steps until obtention of the pellet. Next, parasites were washed twice in 1X PBS pH 7.4 (Gibco, Life Technologies #10010031) and resuspended in 5 mL of lysis buffer (50 mM mono-basic potassium phosphate, 50 mM di-basic potassium phosphate, 0.5 M EDTA, 1 M DTT, 10 mM tosyl-L-lysyl-chloromethane hydrochloride, 0.8% n-octyl-β-d-glucoside, and mini protease inhibitor cocktail, EDTA-free). The subsequent general TPP-TR workflow is summarized in **Figure 1**.

### Cell lysis

To obtain the maximum amount of soluble proteins, cells were subjected to 3 freeze-thaw cycles (liquid nitrogen and water) followed by mechanical shearing. The suspension was then centrifuged at 20,000 × *g* for 20 min at 4°C. Supernatant was collected and quantified using a Bradford assay kit (Thermo Fisher #22662) according to the manufacturer’s recommendations. The protein yield required for a single TPP-TR experiment is 4 mg. Lysate was frozen and stored at -80°C until further use.

### Induction of drug-driven disruption and heat treatment

The lysate was divided into two subsamples: drug-exposed proteins and control (vehicle). The first subsample (drug-exposed) was used for drug-treatment (final concentration of Sb, MF, and AmB: 100 µM), while the other fraction was treated with an equivalent volume of H_2_O. In both conditions, 100 µL of the lysate (≈ 250 µg) was added to seven 0.2-mL microcentrifuge tubes corresponding to seven different temperatures (37, 45, 50, 55, 60, 65 and 70°C). Incubation time was 3 min for each temperature, and conditions with drug or vehicle were run in parallel to induce protein aggregation. Each condition was run in triplicate. After incubation time, the total volume of each tube was transferred to 1.5 mL tubes and centrifuged at 20,000 × *g* for 20 min at 4°C to recover the soluble protein fraction. The supernatant with the soluble proteins was transferred to a new microcentrifuge tube and stored at -80°C for subsequent analyses.

### Collection of soluble protein fraction followed by reduction, alkylation, and digestion

To precipitate soluble proteins, 500 µL of cold acetone was added to each tube. Tubes were incubated for 5 min at room temperature, and then centrifuged at 12,000 × g for 10 min. Proteins within the pellet were then dissolved with 50 mM Tris-HCl (pH = 8). Next, 500 mM DL-dithiothreitol (DTT) solution (Sigma-Aldrich #43816) was added to each sample for a final concentration of 20 mM, mixed briefly, then incubated at 90°C for 10 min. After incubation, proteins were alkylated with 40 mM 2-Iodoacetamide (IAA) (Sigma Life Science #I1149) in darkness at room temperature for 30 min. The alkylation reaction was quenched with the addition of 10 mM of DTT. Finally, trypsin solution was added to the samples at a ratio of 1:20 (protease:protein), followed by a 24 h incubation at 37°C.

### Dimethyl labeling and mass spectrometry

The dimethyl labeling chemistry was previously described by Boersema et al, 2009 (Boersema et al., 2009). Briefly, after 24h incubation with trypsin, samples were dried by vacuum centrifugation for 1 h. Digested samples (250 µg) were reconstituted in 100 µL of 100 mM triethyl ammonium bicarbonate (Sigma-Aldrich). Next, 4 µL of 4% (vol/vol) formaldehyde solution (Sigma Aldrich #252549) and formaldehyde-d_2_ solution (Sigma Aldrich #492620) were mixed into samples now labeled with light (test samples) and heavy dimethyl (internal standard; *Leishmania infantum* WT maintained at 37°C), respectively. Afterward, 4 µL of 0.6 M sodium cyanoborohydride (Aldrich Chemistry #156159) was added to the labeled samples. Solutions were incubated in a fume hood for 1 h at room temperature while mixing with a benchtop test tube mixer. Subsequently, to quench the labeling reaction, 16 µL of 1% (vol/vol) ammonia solution (J.T. Baker) was added and mixed briefly. Finally, 8 µL of formic acid (Sigma Aldrich #27001) was added to further quench the reaction and acidify the sample. Differentially labeled samples were then mixed together for HPLC-MS/MS analysis using a duplex labeling approach.

High-performance liquid chromatography was performed using a Thermo Scientific Vanquish FLEX UHPLC system (San Jose, CA, USA) using gradient elution along with a microbore column (Thermo Biobasic C18 100 × 1 mm, with a particle size of 5 µm). The initial mobile phase condition consisted of acetonitrile and water (both fortified with 0.1% formic acid) at a ratio of 5:95. From 0 to 3 min, the ratio was maintained at 5:95. From 3 to 63 min, a linear gradient was applied up to a ratio of 40:60 and maintained for 2 min. The mobile phase composition ratio was reverted at the initial conditions and the column was allowed to re-equilibrate for 25 min. The flow rate was fixed at 50 µL/min and 5 µL of sample was injected. A Thermo Scientific Q Exactive Plus Orbitrap Mass Spectrometer (San Jose, CA, USA) was interfaced with the UHPLC system using a pneumatic assisted heated electrospray ion source. Nitrogen was used for sheath and auxiliary gases, and they were set at 10 and 5 arbitrary units (a.u.). Auxiliary gas was heated to 200°C. The heated ESI probe was set to 4,000 V and the ion transfer tube temperature was set to 200°C. MS detection was performed in positive ion mode and operating in TOP-10 Data Dependent Acquisition (DDA). A DDA cycle entailed one MS^1^ survey scan (m/z 400-1500) acquired at 70,000 resolution (FWHM) and precursor ions meeting user-defined criteria for charge state (i.e., z = 2, 3 or 4), monoisotopic precursor intensity (dynamic acquisition of MS^2^ based TOP-10 most intense ions with a minimum 2 × 10^4^ intensity threshold) was selected for MS^2^ acquisition. Precursor ions were isolated using the quadrupole (1.5 Da isolation width) and activated by HCD (28 NCE) and fragment ions were detected in the Orbitrap at 17,500 resolution (FWHM). Data were processed using Thermo Proteome Discoverer (version 2.4) in conjunction with SEQUEST, using default settings unless otherwise specified. SEQUEST used a curated database consisting of FASTA sequences extracted from UniProt (i.e., *Leishmania infantum* TAXON ID 5671). The following Proteome Discoverer parameters were set: MS^1^ tolerance of 10 ppm; MS^2^ mass tolerance of 0.02 Da for Orbitrap detection; enzyme specificity was set as trypsin with two missed cleavages allowed; carbamidomethylation of cysteine was set as a fixed modification; dymethylation of lysine and N-terminus was set as a fixed modification and oxidation of methionine was set as a variable modification. Dimethylation 2plex Proteome Discoverer node was used. The minimum peptide length was set to six amino acids, and proteins identified by only one peptide were removed. Data sets were further analysed with Percolator (The et al., 2016). Peptide-spectrum-matches (PSMs) and protein identification were filtered at 1% false discovery rate (FDR) threshold. For protein quantification and comparative analysis, we used the peak integration feature of the Proteome Discoverer 2.4 software (Orsburn, 2021). For each identified protein, the average ion intensity of the unique peptides was used for protein abundance. The complete proteomics dataset is available in **Supplementary Data File 1**. The mass spectrometry proteomics data have been deposited into the ProteomeXchange Consortium *via* the PRIDE (Perez-Riverol et al., 2022) partner repository with the dataset identifier PXD034836 and 10.6019/PXD034836.

### Thermal proteome profiling data processing and normalization

Proteome Discoverer 2.4 software generated the relative abundance of each protein at each temperature. Data was normalized by taking the value of the lowest temperature (37°C) as 1. The generated melting curves were inspected for a change in melting behavior. All melting curves were generated in GraphPad prism (9.3.0) using the following formula:


$$f\left(T\right)=\frac{1-plateau}{1+e^{-\left(\frac{a}{t}-b\right)}}+plateau$$

(Where *T* is the temperature and *a*, *b* and *plateau* are constants (Franken et al., 2015))

Our analysis strategy is based on the comparison of melting points between the various defined conditions (as summarized in **Figure 1**). The temperature at which 50% of the protein is denatured was defined as the melting temperature I. In a drug‐bound state, proteins melt at a higher temperature, thereby causing a thermal shift (*T_m_* drug −*T_m_* vehicle = Δ*T_m_*). The cut-off value for selecting candidate proteins was set at Δ*T_m_* ≥ 4.

### Bioinformatic analysis

Heat maps were generated through the Heat mapper webserver (www.heatmapper.ca/expression) using its protein expression plugin with average linkage as clustering method applied to rows and Euclidean as distance measurement method (Babicki et al., 2016). To identify the pathway of the key proteins selected after thermal shift with the various drugs, an interactome was created using Cytoscape (version 3.9.1) (Shannon et al., 2003). ClueGo was used for pathway enrichment analysis (Bindea et al., 2009). The statistical options for ClueGO enrichment analysis were set based on a two-sided hypergeometric test (*p* ≤ 0.05), Benjamini step down correction, and kappa score ≥ 0.4 (Benjamini and Hochberg, 1995). To predict the signal peptide, the protein sequence was added in the following server: https://services.healthtech.dtu.dk/service.php?SignalP-5.0. To predict transmembrane helices, the protein sequence was added in the server: https://services.healthtech.dtu.dk/service.php?TMHMM-2.0

### Overexpression of candidate genes

Tryparedoxin peroxidase (*TRYP*; *LINF_150018600*), aspartate aminotransferase (*ASP*; *LINF_350012900*) and putative eukaryotic release factor 3 (*ERF3*; *LINF_110017700*) alleles were amplified from genomic DNA derived from *L. infantum* WT parasites using compatible primer pairs (TRYP-Fw: 5’-*Xba*I-ATGTCCTGCGGTGACGC-3’; TRYP-Rv: 5’-*Hind*III- TCACAGCTTGCTGAAGTACC-3’; ASP-Fw: 5’-*Xba*I-ATGTCCACGCAGGCG-3’; ASP-Rv: 5’-*Hind*III-TCACTCGCGATCCAC-3’ and ERF3-Fw: 5’-*XbaI*-ATGTCCTGGCAGCAACCGAC-3’; ERF3-Rv: 5’-*Hind*III-CTACTGGCCGG-3’, respectively). PCR fragments were ligated into pGEM T-easy vector (Promega) to confirm the quality of the insert by Sanger sequencing. Next, PCR fragments were cloned in the *Leishmania* expression vector pSP72α*hyg*α, which contains the gene hygromycin phosphotransferase (*hyg*), a selectable marker in *Leishmania*. A total of 20 μg of plasmid DNA for episomal expression, either the empty vector (mock) or carrying the genes of interest, was then delivered into *L. infantum* WT promastigotes by nucleofection, as previously described (Fernandez-Prada et al., 2018a). Selection was achieved in the presence of a final concentration of 300 μg/mL hygromycin.

### Drug susceptibility assays

Antileishmanial values in promastigotes were determined by monitoring the growth of parasites after 24 h of incubation at 25°C in the presence of increasing concentrations of Sb, by measuring A_600_ using a Cytation 5 multimode reader (BioTek, USA). Drug-efficacy assays were performed with four biological replicates from independent cultures (n = 4). EC_50_ values were calculated based on concentration-response curves analyzed by non-linear regression with GraphPad Prism 9.3.0 software (GraphPad Software, La Jolla California, USA). Statistical analyses were performed using unpaired two-tailed t-tests. A *p* value < 0.05 was considered statistically significant.

## Results

### Impact of antimony on thermal stability of *Leishmania* soluble proteins

**Figure 2:**
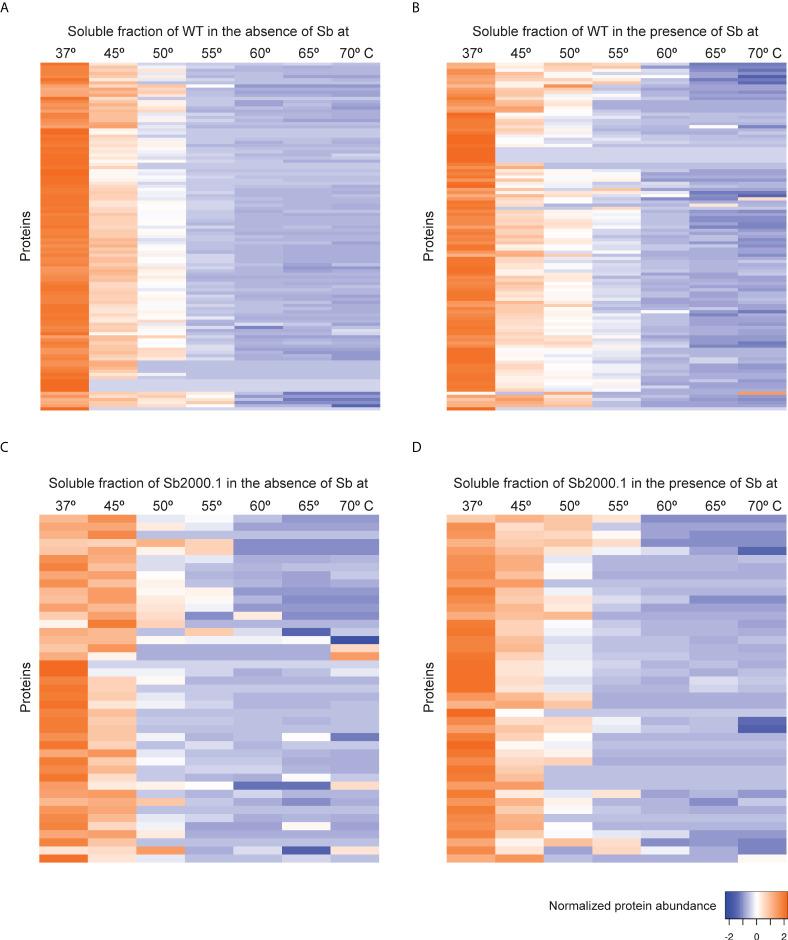
Heat map representation (row Z-score) of the general thermal stability of *Leishmania infantum* (WT and Sb-resistant strains) soluble protein cell extracts. Normalized protein abundance of *L. infantum* WT proteins for which full melting curves were acquired in the absence **(A)** and in the presence **(B)** of 100 µM Sb (111 proteins). Normalized protein abundance of *L. infantum* Sb2000.1 proteins for which full melting curves were acquired in the absence **(C)** and in the presence **(D)** of 100 µM Sb (43 proteins). Color range depicts the relative protein abundance of the soluble fractions at different temperatures. Heat maps were generated through the Heat mapper webserver (www.heatmapper.ca/expression) using its protein expression plugin with average linkage as clustering method applied to rows and Euclidean as distance measurement method.

Although the mechanisms of resistance employed by *Leishmania* against Sb have been extensively described, the MoA of this drug is yet to be completely understood. In order to elucidate Sb targets – as well as potential off-targets – in *Leishmania* parasites, we studied the melting proteome of *L. infantum* Sb-sensitive parasites in the absence and presence of Sb by implementing a novel multiplexed, quantitative TPP-TR approach at a fixed 100 µM drug concentration. To this end, we first acquired quantitative thermal stability data for 1,022 proteins across 7 different temperatures from the *L. infantum* WT cell line (**Supplementary Data File 1**). Among these, we identified 111 proteins showing a temperature-dependent stability variation that allowed the calculation of a melting curve in both untreated and treated experiments (**Figures 2A, B**; **Supplementary Table S1**). As expected, heat maps displaying the global abundance profile of these proteins showed that the general thermal behavior between vehicle (**Figure 2A**) and Sb-treated (**Figure 2B**) samples was modified in the presence of the drug, leading to a greater ligand-binding stabilization of a large number of proteins, especially at lower temperatures (i.e., 37–55°C). As summarized in **Table 1**, we pinpointed 15 soluble proteins showing significant Sb-induced *T_m_* shifts (Δ*T_m_* ≥ 4; according to our significance criteria). Among these potential Sb-binding targets (and off-targets), we identified a tryparedoxin peroxidase (A4HWK2), an aspartate aminotransferase (Q2PD92), a putative eukaryotic release factor 3 (A4HV24), a P-type H^+^-ATPase transmembrane protein (A4HY22), and a hypothetical protein (A4IBA1), as well as several ribosomal proteins (L27, S15, S11, L23a, L36, S21, L10A and L13a) (**Table 1**).

**Table 1:** Summary of proteins identified in Sb-treated and non-treated *Leishmania infantum* WT (sensitive strain) and Sb2000.1 (resistant strain), demonstrating a significant temperature shift.

Accession	Gene ID	Description	*T_m_*_50_ (°C)		Δ*T_m_* (°C)	Signal Peptide (Sec/SPI)^1^	Number of predicted TMHs^2^
			+ Sb	- Sb			
**WT**							
A4I890	*LINF_320033900*	Putative ribosomal protein L27	60.43	46.29	14.14	No	0
A4HWK2	*LINF_150018600*	Tryparedoxin peroxidase	55.81	46.57	9.24	No	0
Q2PD92	*LINF_350012900*	Aspartate aminotransferase	52.68	45.4	7.28	Yes	0
A4HZS1	*LINF_220009800*	40S ribosomal protein S15. putative	54.9	47.63	7.27	No	0
A4HYZ5	*LINF_200021000*	Putative 40S ribosomal protein S11	53.15	47.21	5.94	No	0
A4HSQ5	*LINF_060006500*	Putative Proteasome beta 6 subunit	57.1	51.2	5.9	No	0
A4I2U1	*LINF_270020500*	Putative 60S acidic ribosomal subunit protein P0	50.16	44.32	5.84	No	0
E9AG68	*LINF_060011300*	Putative 60S ribosomal protein L23a	49.81	45.01	4.8	No	0
A4HV24	*LINF_110017700*	Putative eukaryotic release factor 3	45.14	40.4	4.74	No	0
A4IBA1	*LINF_350025300*	Hypothetical protein – conserved	49.4	44.91	4.49	No	0
A4HZ73	*LINF_210013000 *	Putative 60S ribosomal protein L36	49.97	45.59	4.38	No	0
A4HUY7	*LINF_110013400*	Putative 40S ribosomal protein S21	47.51	43.21	4.3	No	0
A4HXT8	*LINF_360046500*	Putative 60S ribosomal protein L10A	51.19	46.92	4.27	No	0
A4HWB9	*LINF_340014400*	Putative 60S ribosomal protein L13a	48.08	43.89	4.19	No	0
A4HY22	*LINF_180020700*	Putative P-type H+-ATPase	51.18	47.03	4.15	No	8
**Sb2000.1**							
A4I4W0	*LINF_290032300*	Putative 60S ribosomal protein L13	50.24	42.54	7.7	Yes	0
A4I574	*LINF_300009700*	Putative aspartyl-tRNA synthetase	49.58	43.8	5.78	No	0
A4I5A6	*LINF_300012900*	Putative 4-methyl-5(beta-hydroxyethyl)-thiazole monophosphate synthesis protein	50.28	45.52	4.76	No	0
A4IB88	* LINF_350023900*	Putative 60S ribosomal protein L5	53.56	49.53	4.03	No	0

1 Sec/SPI: "standard" secretory signal peptides transported by the Sec translocon and cleaved by Signal Peptidase I (Lep) using SignalP - 5.0. 2 Number of predicted transmembrane helices in proteins using TMHMM - 2.0.

Next, we evaluated the impact of Sb in the melting proteome of an *in vitro*-selected *L. infantum* antimony-resistant mutant (Sb2000.1). Again, this experiment was conducted in the absence (**Figure 2C**) and presence of Sb (**Figure 2D**). In total, the Sb2000.1 thermal proteome was comprised of 864 soluble proteins (**Supplementary Data File 1**). Of these, only 43 showed a behavior that allowed for the calculation of melting curves simultaneously in the untreated control and in presence of Sb. Of note, heat maps displaying Sb2000.1 protein abundance profile in the presence of Sb (**Figure 2D**) showed a decreased stabilization (or destabilization) at lower temperatures, and increased ligand-binding stabilization at higher temperatures when compared to the WT exposed to Sb (**Figure 2B**). This, coupled with the reduced number of proteins yielding a melting curve (43 *vs*. 111), points to a generalized decrease in ligand-binding stabilization in the Sb-resistant strain. Of note, only four of the Sb2000.1 proteins for which we were able to calculate a melting curve displayed *T_m_* shifts that fulfilled our significance criteria (**Table 1**): two putative 60S ribosomal proteins (L13 and L5), a putative aspartyl-tRNA synthetase (A4I574), and a putative 4-methyl-5(beta-hydroxyethyl)-thiazole monophosphate synthesis protein (A4I5A6).

**Figure 3:**
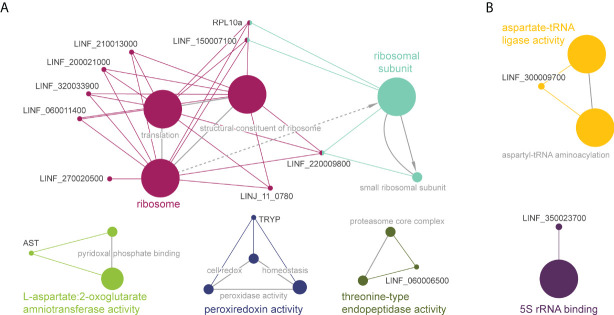
Functional enrichment analysis of proteins with significant *T_m_* shifts in *L. infantum* WT **(A)** and Sb2000.1 **(B)** in the presence of Sb (**Table 1**); created using the ClueGO plugin in the Cytoscape platform. Functionally related clusters of enriched protein sets are represented by different colors, and the label of the most significant term per cluster is shown in bold. The node size represents the term enrichment significance and grey lines, the connectivity (kappa statistics 0.4).

To obtain insight into the potential associations between proteins with significant *T_m_* shifts and define overarching pathways or processes, gene ontology and pathway enrichment analysis were conducted using the ClueGO plugin in Cytoscape (**Figure 3**). In *Leishmania* WT parasites we identified four different clusters (**Figure 3A**). The main cluster consisted of ‘ribosome activity proteins’ (magenta), including ‘translation’ and ‘structural constituents of the ribosome’. This cluster was connected to the ‘ribosomal subunits’ cluster (cyan). The green cluster consisted of ‘aspartate:2-oxoglutarate aminotransferase activity’, and included the aspartate aminotransferase protein (AST), which belongs to the class-I pyridoxal-phosphate-dependent aminotransferase family and mediates the synthesis of L-glutamate – a key amino acid for the synthesis of glutathione (γ-l-glutamyl-l-cysteinylglycine; GSH). The dark blue cluster corresponds to ‘peroxiredoxin activity’, including ‘peroxidase activity’ and ‘cell redox homeostasis’. In this cluster we found a tryparedoxin peroxidase (TRYP), which is a thiol-specific peroxidase involved in the metabolism of trypanothione (T[SH]_2_) – an essential thiol metabolite in maintaining thiol redox homeostasis and controlling oxidative stress in *Leishmania*. The last cluster (olive green) consisted of ‘threonine-type endopeptidase activity’, including the ‘proteasome core complex’. On the other hand, due to the low number of proteins with a Δ*T_m_* ≥ 4 in Sb2000.1, we only identified two minor clusters, corresponding to ‘aspartate-tRNA ligase activity’ (including ‘aspartyl-tRNA aminoacylation) and ‘5S rRNA binding’.

### Impact of miltefosine on thermal stability of *Leishmania* soluble proteins

**Figure 4:**
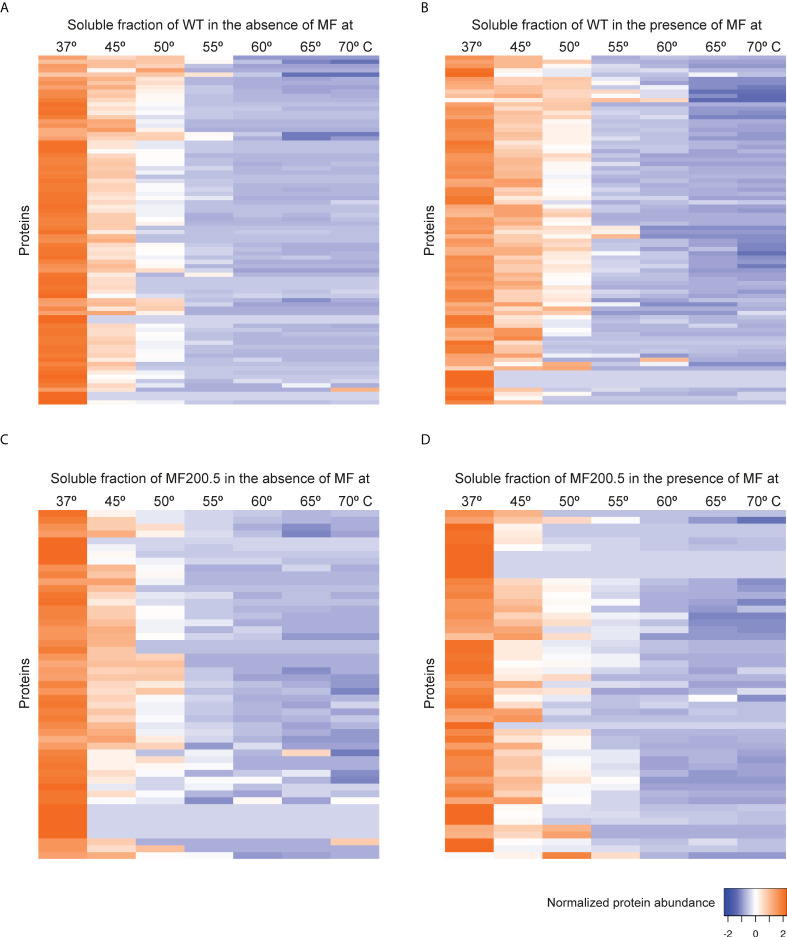
Heat map representation (row Z-score) of the general thermal stability of *Leishmania infantum* (WT and MF-resistant strains) soluble protein cell extracts. Normalized protein abundance of *L. infantum* WT proteins for which full melting curves were acquired in the absence **(A)** and in the presence **(B)** of 100 µM MF (82 proteins). Normalized protein abundance of *L. infantum* MF200.5 proteins for which full melting curves were acquired in the absence **(C)** and in the presence **(D)** of 100 µM MF (51 proteins). Color range depicts the relative protein abundance of the soluble fractions at different temperatures. Heat maps were generated through the Heat mapper webserver (www.heatmapper.ca/expression) using its protein expression plugin with average linkage as clustering method applied to rows and Euclidean as distance measurement method.

Previous studies point to the interaction of MF with lipid metabolism, in addition to glycosylphosphatidylinositol (GPI) anchor biosynthesis and signal transduction in *Leishmania*. To further explore the MoA of MF in *L. infantum*, TPP-TR experiments were conducted first on WT parasites in the absence and in the presence of MF. This led to the identification of 906 soluble proteins, of which 82 displayed a gradient profile in both experimental conditions (**Figures 4A, B**; **Supplementary Table S2**). As previously observed in the TPP-TR Sb experiment, heat maps showing the global abundance profile revealed a highest protein abundance in the presence of the antileishmanial drug (**Figure 4B**) when compared to the untreated control (**Figure 4A**), especially at lower temperatures (i.e., 37–55°C). Next, we explored the melting proteome of the MF200.5 strain (MF-resistant counterpart), leading to the identification of 901 proteins present in both drug-unexposed and drug-exposed proteomes (**Supplementary Data File 1**). We were then able to generate melting curves for 51 of these proteins (**Figures 4C, D**). Of note, the MF200.5 strain showed lower protein stabilization than the WT, both in the absence (**Figure 4C**) and presence of MF (**Figure 4D**), pointing to reduced interaction of MF with the proteome of the MF-resistant strain.

Next, we identified 20 proteins in the WT and 5 proteins in MF200.5 displaying *T_m_* shifts that fulfilled our significance criteria (**Table 2**). Among WT proteins with a Δ*T_m_* ≥ 4 we identified: two tRNA synthetases (threonyl and isoleucyl), several ribosomal proteins (L24, L34 S2, S4, S15 and S18), a phosphotransferase (A4HZB2), a malic enzyme (A4I0R5), a fumarate hydratase (A2CIN2), and a citrate synthase (A4HXU5). On the other hand, the 5 significant proteins found in the MF200.5 melting proteome included a centromere/microtubule binding protein cbf5 (A4HZI2), an R-SNARE protein (A4I839), a GMP–DE - delta subunit (A4I307), a 40S ribosomal protein S5 (A4HV05), and a putative small myristoylated protein-1 (A4HYX4).

**Table 2:** Summary of proteins identified in MF-treated and non-treated *Leishmania infantum* WT (sensitive strain) and MF200.5 (resistant strain), demonstrating a significant temperature shift.

Accession	Gene ID	Description	*T_m_*_50_ (°C)		Δ*T_m_* (°C)	Signal Peptide (Sec/SPI)^1^	Number of predicted TMHs^2^
			+ MF	- MF			
**WT**							
A4IB34	*LINF_350019000*	Putative threonyl-tRNA synthetase	52.1	38.4	13.8	No	0
A4ICM4	*LINF_360016500*	Putative ribosomal protein L24	49.7	40.4	9.3	No	0
A4I0R5	*LINF_240012700*	Malic enzyme	48.5	40.3	8.2	No	0
A4HZB2	*LINF_210007900*	Phosphotransferase	55.3	47.6	7.7	No	0
A4IDD6	*LINF_360022900*	Proteasome subunit beta type-5	59.9	52.8	7.1	No	0
A4ICP1	*LINF_360015100*	Putative 40S ribosomal protein S18	52.0	45.6	6.3	No	0
A4I7P2	*LINF_320013000*	NRBD1 protein	52.1	46.3	5.9	No	0
A2CIN2	*LINF_290027200*	Fumarate hydratase	51.0	45.2	5.8	No	0
A4HXU5	*LINF_180012200*	Citrate synthase	54.0	48.3	5.7	No	0
A4HV09	*LINF_110016000*	Pyruvate, phosphate dikinase	48.2	42.5	5.7	No	0
E9AGQ8	*LINF_190005400*	40S ribosomal protein S2	51.4	46.0	5.4	No	0
A4IDX6	*LINF_360066000*	Putative isoleucyl-tRNA synthetase	47.9	42.6	5.3	No	0
A4I9A6	*LINF_330035900*	CCHC-type domain-containing protein	50.1	44.9	5.2	No	0
A4I9F0	*LINF_330041000*	Uncharacterized protein	55.34	50.58	4.76	No	0
A4I973	*LINF_330026200*	Uncharacterized protein	49.64	45.01	4.63	No	0
E9AHW1	*LINF_350027200*	DRBD2 protein	49.03	44.62	4.41	No	0
A4HZS1	*LINF_220009800*	Putative 40S ribosomal protein S15	48.32	43.98	4.34	No	0
A4HX73	*LINF_170007200*	Elongation factor 1-alpha	49.59	45.45	4.14	No	0
A4HVQ1	*LINF_130017300*	Putative 40s ribosomal protein s4	49.24	45.19	4.05	No	0
A4HY10	*LINF_180019400*	Putative 60S ribosomal protein L34	50.47	46.46	4.01	No	0
**MF200.5**							
A4HZI2	*LINF_210027400*	Centromere/microtubule binding protein cbf5 - putative	51.1	40.5	10.6	No	0
A4I839	*LIN_320027900*	R-SNARE protein - putative	53.6	46.4	7.1	No	0
A4I307	*LINF_270027100*	GMP-PDE - delta subunit - putative	54.1	47.4	6.7	No	0
A4HV05	*LINF_110015600*	40S ribosomal protein S5	51.6	45.8	5.8	No	0
A4HYX4	*LINF_200018300 *	Putative small myristoylated protein-1	49.2	43.9	5.3	No	0

1 Sec/SPI: "standard" secretory signal peptides transported by the Sec translocon and cleaved by Signal Peptidase I (Lep) using SignalP - 5.0. 2 Number of predicted transmembrane helices in proteins using TMHMM - 2.0

**Figure 5:**
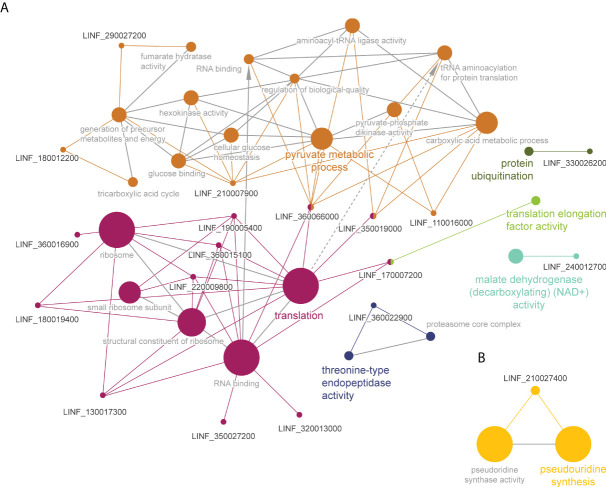
Functional enrichment analysis of proteins with significant *T_m_* shifts in *L. infantum* WT **(A)** and MF200.5 **(B)** in the presence of MF (**Table 2**); created using the ClueGO plugin in the Cytoscape platform. Functionally related clusters of enriched protein sets are represented by different colors and the label of the most significant term per cluster is shown in bold. The node size represents the term enrichment significance and grey lines, the connectivity (kappa statistics 0.4).

Gene ontology and pathway enrichment analysis of these two subsets of significant proteins led to the identification of six pathways in the WT (**Figure 5A**) and one in the MF-resistant strain (**Figure 5B**). The WT melting proteome’s enriched pathways were comprised of: (i) a major cluster grouping proteins belonging to the ‘pyruvate metabolic process’ (orange), which was connected to the (ii) ‘translation’ (magenta) and (iii) ‘translation elongation activity’ (green) clusters; (iv) ‘protein ubiquitination’ (olive green); (v) ‘malate dehydrogenase activity’ (cyan) and; (vi) ‘threonine-type endopeptidase activity’. By contrast, ‘pseudouridine synthase activity’ was the only pathway/activity identified for the 5-protein set of the MF-resistant strain.

### Effect of amphotericin B on the meltome of *L. infantum*

**Figure 6:**
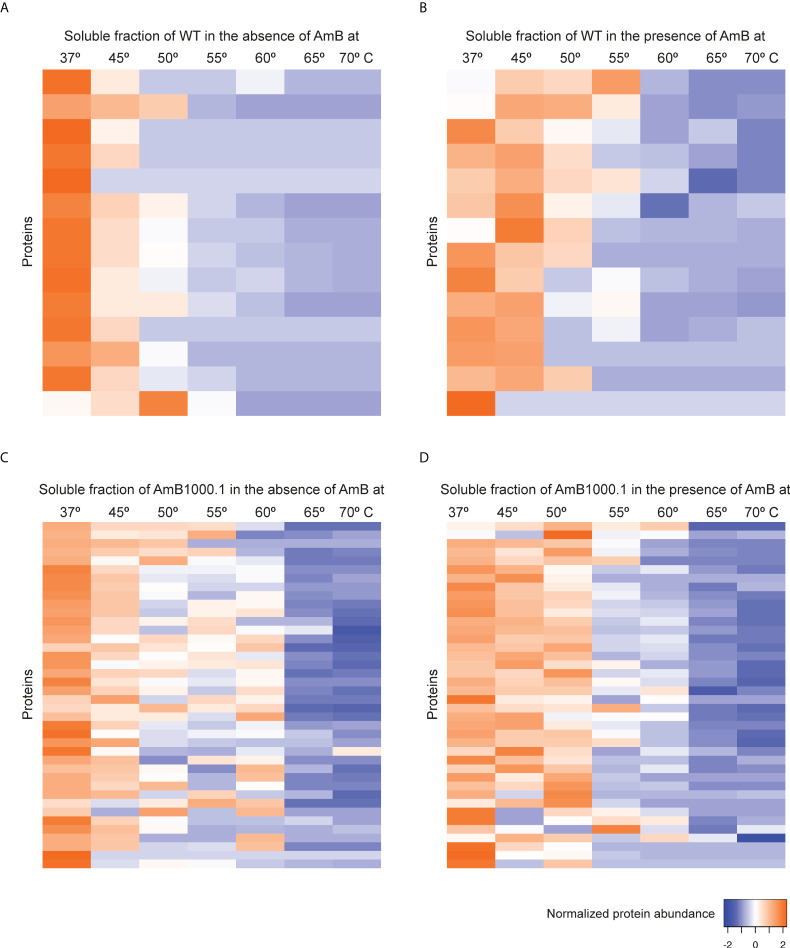
Heat map representation (row Z-score) of the general thermal stability of *Leishmania infantum* (WT and AmB-resistant strains) soluble protein cell extracts. Normalized protein abundance of *L. infantum* WT proteins for which full melting curves were acquired in the absence **(A)** and in the presence **(B)** of 100 µM AmB (14 proteins). Normalized protein abundance of *L. infantum* AmB1000.1 proteins for which full melting curves were acquired in the absence **(C)** and in the presence **(D)** of 100 µM AmB (40 proteins). Color range depicts the relative protein abundance of the soluble fractions at different temperatures. Heat maps were generated through the Heat mapper webserver (www.heatmapper.ca/expression) using its protein expression plugin with average linkage as clustering method applied to rows and Euclidean as distance measurement method.

The main known MoA of AmB in *Leishmania* appears to be a disruption of the permeability barrier of the parasite by targeting membrane sterols. Following the same principle of Sb and MF, we explored AmB’s potential direct and indirect targets using an AmB-sensitive/resistant pair of *L. infantum* strains, in the presence and absence of the antileishmanial agent. TPP-TR led to the acquisition of quantitative thermal stability data for 946 proteins across 7 different temperatures from the *L. infantum* WT cell line, and 836 proteins from the AmB-resistant mutant (**Supplementary Data File 1**). We were able to generate 14 melting curves from the data of the WT in the absence (**Figure 6A**, **Supplementary Table S3**) and in the presence of AmB (**Figure 6B**), and 40 for the AmB-resistant strain (**Figures 6C, D**, **Supplementary Table S3**). As depicted in **Figure 6**, the presence of AmB led to a marked stabilization of the melting proteome WT up to 55°C (**Figure 6B**). This phenomenon was not observed in the AmB-resistant strain, which showed similar levels of stabilization in the presence and the absence of AmB (**Figure 6D**).

Calculation of the melting curves led to the identification of 9 proteins in the WT and 12 proteins in AmB1000.1 displaying *T_m_* shifts that fulfilled our significance criteria (**Table 3**). Among WT proteins with a Δ*T_m_* ≥ 4 we identified: a putative protein belonging to the Rieske complex (A4IB55), heat-shock protein 83 (E9AHM9), a thiol-specific peroxidase involved in cellular oxidant detoxification (Q95U89), an adenosylhomocysteinase (A4ID05) responsible for the synthesis of L-homocysteine, and a putative N(alpha)-terminal acetyltransferase (A4HXH5). On the other hand, the 12 significant proteins found in the AmB1000.1 melting proteome included two ribosomal proteins (L36 and S5), a chaperonin CCT-alpha (A4I8F2), a putative ALBA3 protein (A4IA34) – known to be involved in regulating gene expression during parasite development –, and a glucose-6-phosphate isomerase (A0A142BXU6) involved in the synthesis of D-glyceraldehyde.

**Table 3:** Summary of proteins identified in AmB-treated and non-treated *Leishmania infantum* WT (sensitive strain) and AmB1000.1 (resistant strain), demonstrating a significant temperature shift.

Accession	Gene ID	Description	*T_m_*_50_ (°C)		Δ*T_m_* (°C)	Signal Peptide (Sec/SPI)^1^	Number of predicted TMHs^2^
			+ AmB	- AmB			
**WT**							
A4IB55	*LINF_350020400*	Rieske iron-sulfur protein - mitochondrial precursor - putative	60.8	48.3	12.5	No	1
E9AHM9	*LINF_330009000*	Heat shock protein 83-1	56	45	11	No	0
A4HZS1	*LINF_220009800*	Putative 40S ribosomal protein S15	57.2	46.6	10.6	No	0
Q95U89	*LINF_230005400*	Peroxiredoxin	54.8	45.1	9.8	No	0
A4ID05	*LINF_360048000*	S-adenosylhomocysteine hydrolase	59.6	51.3	8.3	No	0
A4IE56	*LINF_360050900*	Oxidoreductase - putative	50.8	44.4	6.4	No	0
A4HYZ5	*LINF_200021000*	Putative 40S ribosomal protein S11	53.3	47.1	6.2	No	0
A4HV47	*LINF_120005200*	proteasome beta-1 subunit - putative	59.97	55.37	4.6	No	0
A4HXH5	*LINF_170016800*	N(alpha)-terminal acetyltransferase - putative	44.54	40.33	4.21	No	0
**AmB1000.1**							
A4HZ73	*LINF_210013000*	Putative 60S ribosomal protein L36	60.5	41.8	18.7	No	0
A4I8F2	*LINF_320040200*	Chaperonin alpha subunit - putative	68.7	55.1	13.6	No	0
A4I357	*LINF_270006800*	Proteasome alpha 7 subunit - putative	65.3	57.5	7.8	No	0
A4IA34	*LINF_340031800*	Alba3 - putative	55.9	48.4	7.5	No	0
A0A142BXU6	*LINF_120010600*	Glucose-6-phosphate isomerase	55	48	6.9	No	0
A4IB31	*LINF_350018700*	Putative mitochondrial processing peptidase - beta subunit	53.4	46.6	6.8	No	0
A4HV05	*LINF_110015600*	40S ribosomal protein S5	69.9	63.2	6.7	No	0
A4ICW8	*LINF_360007100*	Elongation factor 2	57.4	51	6.4	No	0
A0A0S2UQ61	*LINF_280034700*	Activated protein kinase C receptor	50.1	44.1	6.1	No	0
A4I9P1	*LINF_340014000*	Elongation factor 1-beta	50.5	44.9	5.6	No	0
A4HUJ7	*LINF_100015200*	Putative nuclear transport factor 2	55.9	50.9	5.1	No	0
A4IB34	* LINF_350019000*	Putative threonyl-tRNA synthetase	50.04	45.32	4.72	No	0

1 Sec/SPI: "standard" secretory signal peptides transported by the Sec translocon and cleaved by Signal Peptidase I (Lep) using SignalP - 5.0. 2 Number of predicted transmembrane helices in proteins using TMHMM - 2.0.

**Figure 7:**
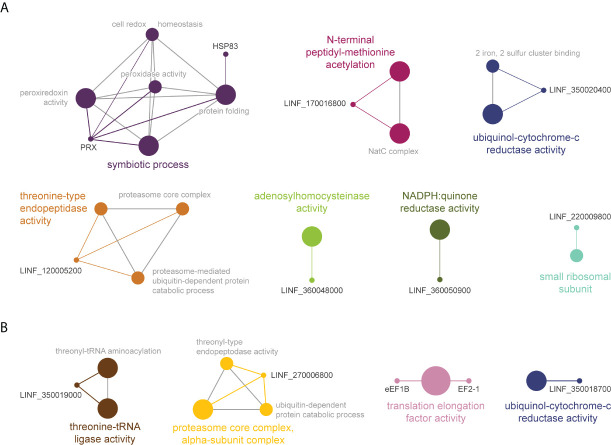
Functional enrichment analysis of proteins with significant *T_m_* shifts in *L. infantum* WT **(A)** and AmB1000.1 **(B)** in the presence of AmB (**Table 3**); created using the ClueGO plugin in the Cytoscape platform. Functionally related clusters of enriched protein sets are represented by different colors and the label of the most significant term per cluster is shown in bold. The node size represents the term enrichment significance and grey lines, the connectivity (kappa statistics 0.4).

Gene ontology and pathway enrichment analysis classified AmB TPP-TR significant proteins in 7 pathways (**Figure 7A**). The largest cluster corresponded to ‘symbiotic process[es]’ (purple), including ‘cell redox homeostasis’, as well as ‘peroxiredoxin and peroxidase activities’, potentially responsible for controlling oxidative stress induced by AmB. The other pathways identified were: (i) ‘threonine-type endopeptidase activity’ pathway (orange), including proteasome-related processes; (ii) ‘N-terminal peptidyl-methionine acetylation’ (magenta); (iii) ‘ubiquitinol-cytochrome c reductase activity’ (blue); (iv) ‘adenosylhomocysteinase activity’ (green); (v) ‘NADPH:quinone reductase activity’ (olive green) and; (vi) ‘small ribosomal subunit’ (cyan). Interestingly, the ‘proteasome-related pathway’ (yellow) and ‘ubiquitinol-cytochrome c reductase activity’ (dark blue) were also among the pathways enriched in the meltome of AmB1000.1 (**Figure 7B**). Two other pathways were also identified, corresponding to ‘threonine-tRNA ligase activity’ and ‘translation elongation factor activity’.

### Impact of candidate genes on susceptibility of *L. infantum* to antimony

**Figure 8:**
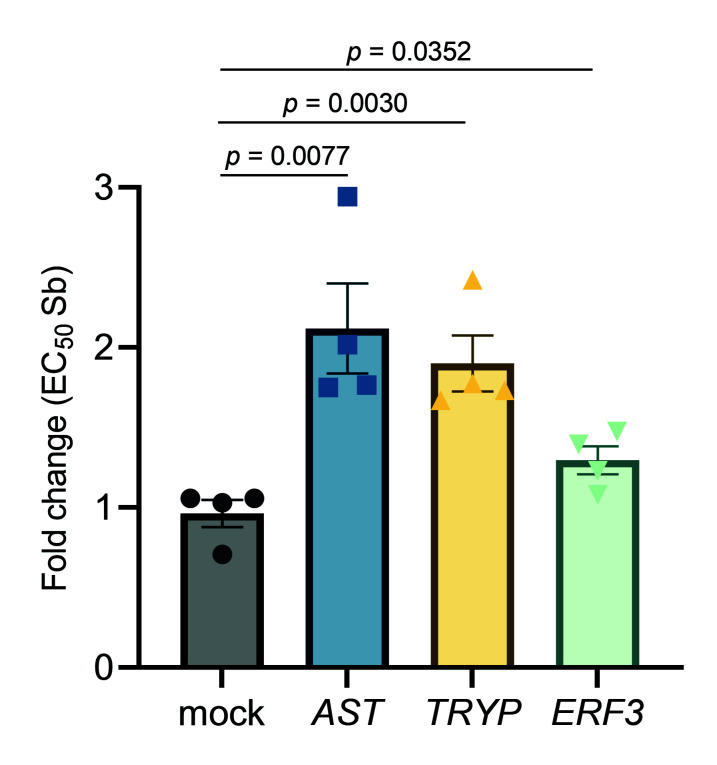
Impact of overexpression of the leishmanial tryparedoxin peroxidase (*TRYP*), aspartate aminotransferase (*AST*), and putative eukaryotic release factor 3 (*ERF3*) genes on the Sb sensitivity profile of *L. infantum*. EC_50_ values were calculated from concentration–response curves performed in quadruplicate after nonlinear fitting. Data are the mean ± SEM of four biological replicates.

The profiling of targeted and off-target drug-protein binding could represent a major application of TPP-TR in discovering markers of drug resistance in *Leishmania* parasites. However, it is important to confirm that modulation of the identified target is associated with functional effects that are detectable in phenotypic assays. As a proof of principle, we further evaluated the potential impact of three candidates, non-ribosomal proteins tryparedoxin peroxidase (*TRYP*; Δ*T_m_* = 9.24), aspartate aminotransferase (*AST*; Δ*T_m_* = 7.28) and putative eukaryotic release factor 3 (*ERF3*; Δ*T_m_* = 4.74), on *Leishmania*’s susceptibility to Sb. Our preferred method for studying their role was the episomal transfection of the three genes of interest into the *L. infantum* WT background. As depicted in **Figure 8**, overexpression of all three Sb-targeted candidate genes resulted in a significant decrease in Sb sensitivity in the WT background: *AST* (2.12-fold; *p =* 0.0077), *TRYP* (1.90-fold; *p =* 0.0030) and *ERF3* (1.30-fold; *p =* 0.0352), when compared to the mock-transfected *Leishmania* line.

## Discussion

One of the major bottlenecks in drug discovery is the identification of the molecular targets of a compound (Fernández-Prada et al., 2019; Cohen and Azas, 2021), which, if not fully elucidated, hinders subsequent structure-activity relationship studies that have the potential to contribute to drug optimization and drug development (Terstappen et al., 2007). While proteomic-based approaches have become a cornerstone in the study of MoA and MoDR in *Leishmania* parasites, there remain important knowledge gaps to fill before these mechanisms are holistically understood (Fernandez-Prada et al., 2018b; Bhattacharya et al., 2020; Douanne et al., 2020a). In this work we have implemented a novel TPP-TR multiplexed method to further identify and classify the various pathways targeted by current antileishmanial drugs and their MoA. This approach led to the identification and validation of novel Sb target/resistance proteins, as well as highlighting several pathways potentially involved in the MoA of MF and AmB.

Using the antileishmanial drug Sb as initial proof of concept, we generated the first ‘meltome’ of a sensitive/resistant pair of *Leishmania* strains in the absence and in the presence of the drug. Analysis of the melting curves of the cell-extracted proteome led to the identification of several proteins with altered thermal stability in the presence of Sb. The mechanism of killing of reduced antimony (Sb^III^) is suspected to involve trypanothione reductase (TR) and CCHC/CCCH-type zinc-finger protein as potential molecular targets through binding of the drug to the deprotonated thiol group of the cysteine residue (Cys binding) (Haldar et al., 2011; Frezard et al., 2012). Trivalent antimonials interfere with T(SH)_2_ metabolism by inhibiting TR and inducing rapid efflux of intracellular T(SH)_2_ and GSH (Wyllie et al., 2004). Of note, our TPP-TR Sb experiments highlighted the ‘aspartate:2-oxoglutarate aminotransferase activity’ pathway as highly enriched, including AST and TRYP, two major enzymes involved in maintaining thiol redox homeostasis and controlling oxidative stress in *Leishmania* (Turcano et al., 2018). TRYP displayed the second highest Δ*T_m_* value, pointing to high-affinity binding between Sb and TRYP that could correlate with potential TRYP druggability by Sb. Overexpression of a drug’s molecular target often increases drug resistance, offering a tool for target identification. Episomal overexpression of TRYP led to a 1.9-fold reduction in Sb sensitivity in the *L. infantum* WT background, probably as a direct result of increasing the basal amount of drug’s target. Our findings further corroborate previous studies associating the overexpression of TRYP with resistance to Sb in both *in vitro*-generated *Leishmania* resistant lines (Wyllie et al., 2008) and field isolates (Wyllie et al., 2010). Our TPP-TR experiments also highlighted the thermal stabilization of AST in the presence of Sb, with a Δ*T_m_* very close to that of TRYP. Of note, AST is an essential component in the biosynthetic pathways of polyamines and GSH by catalyzing the conversion of aspartate and alpha-ketoglutarate to oxaloacetate and L-glutamate, which is essential in the metabolism of T(SH)_2_. Overexpression of AST resulted in an increased protection (i.e., 2.1-fold increase) against Sb, further confirming our TPP-TR Sb stabilization results. Of note, our experiments were conducted on cell extracts where ligand binding but no downstream or feedback effects occur (Savitski et al., 2014). This limitation could be solved in future experiments exploring *T_m_* shifts in intact parasites, further elucidating the impact of the binding of Sb to AST on downstream TRYP’s activity, and vice versa.

Our analyses revealed various ribosomal proteins showing a significant thermal stabilization when exposed to Sb. Metal ions are an integral component of RNA molecules, and they contribute to the structural stability of the large subunit of eukaryotic ribosomes (Klein et al., 2004). It is known that metal ions other than Mg^2+^, including Tb^3+^, can displace Mg^2+^ from its high-affinity binding sites (Hargittai and Musier-Forsyth, 2000). While Sb can bind to and interact with nucleic acids (Lai et al., 2022), it is not clear whether trivalent antimony could displace Mg^2+^. Our hypothesis is that ribosome-RNA complexes serve as ‘off-target traps’ in which Sb binds to RNA. Among the ribosomal proteins pinpointed in our experiments, we noted the 60s ribosomal L23a protein (Δ*T_m_* = 4.8) as a potential target of Sb. Interestingly, this protein is potentially involved in lowering and redistributing Sb pressure in Sb-resistant clinical isolates; its overexpression decreases the sensitivity of sensitive parasites to Sb (Das et al., 2013). This protein has recently been shown to be phosphorylated by a calcium-dependent protein kinase 1 (CDPK1) which is able to modulate translation efficiency. Markedly, mutations in CDPK1 contribute to paromomycin and Sb resistance (Bhattacharya et al., 2019). Our analyses also pointed to another member of the translation pathway: ERF3, an essential GTPase responsible for cleavage of the peptide bond as well as for the release of ribosomal subunits in eukaryotes (Alkalaeva et al., 2006). Recent immunoprecipitation studies have revealed major interactions of CDPK1 with structural constituents of the ribosome and GTPase activity proteins (Bhattacharya et al., 2019). Overexpression of ERF3 in the WT background led to a slight, but significant, decrease in sensitivity to Sb. Further work could reveal if there are any direct interactions between ERF3 and Sb, or if this potential ligand-binding stabilization involves RNA as a direct target. Altogether, evidence points to translation mediators as indirect targets of Sb. In this way, modulation of translation levels could represent a general mechanism to counter the effect of Sb, and probably that of other antileishmanials – we also identified several ribosomal proteins and RNA-binding proteins with high *T_m_* shifts in MF and AmB experiments (**Tables 2**, **3**).

Next, we explored the meltome of *L. infantum* WT, and its MF- and AmB-resistant counterparts, in the presence of MF and AmB. MF is known to interact with lipids, inhibiting cytochrome-c oxidase and leading to mitochondrial dysfunction and programmed cell death (Lux et al., 1996). TPP-TR revealed an overrepresentation of proteins belonging to the tricarboxylic acid (TCA) cycle and the gluconeogenesis pathways – fatty acids are substrates for both pathways – when cell extracts were exposed to alkyl-phospholipid drug MF. Downregulation of drug target expression can potentially lead to drug-resistant phenotypes. In this way, a mitochondrial proteomic comparative analysis revealed the downregulation of several key enzymes related to the TCA cycle in MF-resistant parasites (Vincent et al., 2015). Among the other proteins potentially interacting with MF, we found a previously uncharacterized protein (*LINF_330026200*) that could be involved in glycosylphosphatidylinositol (GPI)-anchor biosynthesis (KEGG ec00563), further supporting previous reports on the subject of MF’s MoA (Lux et al., 1996). Interestingly, in the presence of MF, we identified a SNARE protein in the meltome of MF200.5. Likewise, a recent work by Garcia et al. (2021) highlighted the potential role of SNARE-mediated vesicle transport in MF resistance in *Saccharomyces cerevisiae* (Garcia et al., 2021). Moreover, a putative small myristoylated protein-1 (SMP-1) was also stabilized in the presence of MF, pointing to an enhanced ER-Golgi-mediated trafficking towards the flagellum in the MF-resistant line (Tull et al., 2010). Markedly, in one of our recent studies, we identified a differential enrichment of SMP-1 in exosomes released by MF200.5 (Douanne et al., 2020a). Of note, *Leishmania* secretes exosomes through multivesicular bodies and flagellar pockets (Atayde et al., 2015). Future work could reveal if extracellular vesicles serve as a pathway for sequestration of MF, reducing the effective concentration at target sites (MoDR). While not fully elucidated, the MoA of AmB involves the generation of channel-like pores spanning the lipid bilayer, by binding of AmB preferentially to membrane ergosterol, leading to cell death (Mbongo et al., 1998; Pourshafie et al., 2004). The presence of AmB led to a great stabilization of heat shock protein 83 (HSP83, also referred as HSP90), a chaperone, in our TPP-TR experiments. Chaperones are central players controlling stress-related processes in *Leishmania*. Cytosolic HSP83 is involved in protein maturation of steroid receptors (Requena et al., 2015). Interestingly, HSP83 did not display thermal stabilization in the AmB-resistant line. Recent studies have shown that the levels of ergosterol and 5-dehydroepisterol are greatly decreased in AmB1000.1 (Fernandez-Prada et al., 2016). Moreover, HSP83 increased drug resistance and reduced drug-mediated activation of programmed cell death by interfering with mitochondrial membrane potential (Vergnes et al., 2007). In mammals, for mitochondrial targeting, preproteins associate with both HSP90 and HSP70 in the cytosol (Young et al., 2003). Our analysis identified the mitochondrial precursor of a Rieske Fe-S protein (RIP-1) subunit of the cytochrome c reductase complex – with a similar Δ*T_m_* to HSP83. Further studies could reveal whether HSP83 is involved in RIP-1 translocation and how these are directly or indirectly targeted by AmB. We also identified two other potential AmB targets, peroxiredoxin (PRX) and S-adenosylhomocysteine hydrolase (SAHH). PRX exerts intrinsic ATP-independent chaperone activity in *L. infantum*, protecting a wide variety of proteins from heat stress-mediated unfolding (Teixeira et al., 2015). Moreover, PRX proteins are Cys-based peroxidases that act as mechanism of defense for detoxification of reactive oxygen species in several organisms (i.e., bacterial, and fungal pathogens). Markedly, targeted replacement of the *prx1* gene led to a slight increase in sensitivity against AmB and SDS in *Aspergillus fumigatus* (Rocha et al., 2018). On the other hand, SAHH synthesizes L-homocysteine, an essential precursor for terpenoid backbone biosynthesis (i.e., ergosterol). Activation of alternative pathways would explain previous studies reporting reduced levels of ergosterol in AmB1000.1, which translates to a lower affinity of AmB for AmB-resistant parasite membranes (Fernandez-Prada et al., 2016). Selection of stress response-associated proteins through TPP-TR upon AmB or MF treatment could point to “innate” strategies used by *L. infantum* to survive in stressful environments. It is also worthy to note that in AmB and MF TPP-TR experiments we were able to detect a significant *T_m_* shift for various components of the proteasome, whose inhibition is known to trigger proteotoxic stress in the parasite (Bijlmakers, 2020). Curiously, the TPP-TR approach also highlighted proteins previously reported to elicit protective immunity against VL, such as EF-1alpha and HSP83 (Kaur et al., 2011; Sabur et al., 2018).

Although we were able to pinpoint previously known drug targets, as well as identify novel ones, there are still various limitations in our study. In our context, only very few targets harboring transmembrane helical segments were selected through the TPP-TR approach, confirming that it’s more suitable for soluble proteins. Because of this, some important membrane proteins that are not solubilized under our experimental conditions (i.e., aquaglyceroporin-1 (AQP1) in Sb trafficking (Monte-Neto et al., 2015)) might have gone undetected. Moreover, there are some proteins that do not significantly change their thermal stability upon ligand binding, thus precluding their identification as direct or indirect targets through our method. Although overexpression of proteins displaying a significant T*m* shift led to a gain-of-function resistance phenotype in certain cases (i.e., TRYP, AST and ERF3), TPP-TR fails to identify potential loss-of-function mechanisms (i.e., MF transporter mutations in MF and AmB resistance (Fernandez-Prada et al., 2016) or AQP1 mutations in Sb resistance (Monte-Neto et al., 2015)) that could be potentially involved in other MoDR. Additionally, interactions with small molecules or proteins, or even post‐translational modifications, could lead to T*m* shifts that are not directly related to drug-binding. Finally, although experiments were performed in biological triplicates, instead of treating the replicates as an individual sample, data were treated through pooling of the biological replicates. This may therefore lead to some limitations with respect to extrapolation of our data.

In conclusion, we generated the first meltome of *L. infantum* in the absence and in the presence of three main antileishmanial drugs, leading to the identification and validation of novel drug targets. We also provided a complete list of metabolic pathways that could be directly or indirectly linked to MoA/MoDR, pinpointing numerous potential targets that could serve as a jumping-off point for developing single or combinatory on-target treatments based on novel and/or repurposed drugs.

## Data availability statement

The datasets presented in this study can be found in online repositories. The names of the repository/repositories and accession number(s) can be found in the article/**Supplementary Material**.

## Author contributions

Conceptualization, AI-M, FB, CF-P. Methodology, AI-M, FB, CF-P. Investigation, AI-M, AC, VW, FB. Formal analysis, AI-M, FB, RM-N, CF-P. Writing – original draft, AI-M, AC, VW. Writing – review and editing, FB, RM-N, CF-P. Visualization, AI-M, FB, CF-P. Supervision, FB, CF-P. Resources, FB, RM-N, CF-P. Funding Acquisition, FB, CF-P. All authors contributed to the article and approved the submitted version.

## Funding

Work in the CFP-Lab was supported by a Natural Sciences and Engineering Research Council of Canada (NSERC) Discovery Grant RGPIN-2017-04480 and by the Canada foundation for Innovation (www.innovation.ca), grant numbers 37324 and 38858. AC is supported by an Alexander-Graham-Bell PhD NSERC scholarship. The proteomic investigations were funded by the National Sciences and Engineering Research Council of Canada (F. Beaudry discovery grant no. RGPIN-2020-05228). Laboratory equipment was funded by the Canadian Foundation for Innovation (F. Beaudry CFI John R. Evans Leaders grant no. 36706). FB is the holder of the Canada Research Chair in metrology of bioactive molecule and target discovery (grant no. CRC-2021-00160).

## Acknowledgments

Authors want to thank Prof. Marc Ouellette for the kind gift of the *L. infantum* Sb2000.1, MF200.5 and AmB1000.1 drug-resistant strains. We also thank Dr. Aida Mínguez-Menéndez for her help with the creation of scientific figures.

## Conflict of interest

The authors declare that this research was conducted in the absence of any commercial or financial relationships that could be construed as a potential conflict of interest.

## Publisher’s note

All claims expressed in this article are solely those of the authors and do not necessarily represent those of their affiliated organizations, or those of the publisher, the editors and the reviewers. Any product that may be evaluated in this article, or claim that may be made by its manufacturer, is not guaranteed or endorsed by the publisher.
