# Supplementary material for: Exploring direct and indirect targets of current antileishmanial drugs using a novel thermal proteomics profiling approach
Source: Front Cell Infect Microbiol. 2022 Aug 3;12:954144. doi: 10.3389/fcimb.2022.954144 (PMC9381709; doi:10.3389/fcimb.2022.954144)
Supplement: Supplementary file 2 [file Table_1.docx]

Supplementary Material

# Supplementary Table

**Supplementary Table 1.** Gene ID of the proteins identified in *L.infantum* WT strain (111 proteins) and Sb-resistant strains (43 proteins). The order of the proteins in each condition represents the protein's order that appears in the heat map for antimony.

| **WT** | |  | **WT+ Sb** | |  | **Sb2000.1** | |  | **Sb2000.1+Sb** | |
| --- | --- | --- | --- | --- | --- | --- | --- | --- | --- | --- |
| order | Gen ID |  | order | Gen ID |  | order | Gen ID |  | order | Gen ID |
| 43 | LINF_350038800 |  | 103 | LINF_350052900 |  | 28 | LINF_100015200 |  | 40 | LINF_010009900 |
| 18 | LINF_150018600 |  | 35 | LINF_360015100 |  | 20 | LINF_250027000 |  | 30 | LINF_050008500 |
| 105 | LINF_360063600 |  | 75 | LINF_050009500 |  | 41 | LINF_130022300 |  | 35 | LINF_270006800 |
| 12 | LINF_240027300 |  | 29 | LINF_350023900 |  | 37 | LINF_120005200 |  | 34 | LINF_360022900 |
| 59 | LINF_350021700 |  | 25 | LINF_360015700 |  | 34 | LINF_360022900 |  | 16 | LINF_230007100 |
| 17 | LINF_090016000 |  | 82 | LINF_200021000 |  | 13 | LINF_360081500 |  | 4 | LINF_360048000 |
| 108 | LINF_350056600 |  | 58 | LINF_230005400 |  | 4 | LINF_360048000 |  | 3 | LINF_250018000 |
| 82 | LINF_200021000 |  | 46 | LINF_300009700 |  | 3 | LINF_250018000 |  | 37 | LINF_120005200 |
| 11 | LINF_350005300 |  | 59 | LINF_350021700 |  | 12 | LINF_330034600 |  | 41 | LINF_130022300 |
| 5 | LINF_360048000 |  | 23 | LINF_330034600 |  | 35 | LINF_270006800 |  | 12 | LINF_330034600 |
| 75 | LINF_050009500 |  | 71 | LINF_040009700 |  | 30 | LINF_050008500 |  | 28 | LINF_100015200 |
| 99 | LINF_320027900 |  | 27 | LINF_100007300 |  | 40 | LINF_010009900 |  | 20 | LINF_250027000 |
| 54 | LINF_160009700 |  | 108 | LINF_350056600 |  | 26 | LINF_130021300 |  | 17 | LINF_060006500 |
| 29 | LINF_350023900 |  | 79 | LINF_270027100 |  | 17 | LINF_060006500 |  | 33 | LINF_200018300 |
| 14 | LINF_340035600 |  | 90 | LINF_350019000 |  | 16 | LINF_230007100 |  | 22 | LINF_230005400 |
| 79 | LINF_270027100 |  | 12 | LINF_240027300 |  | 6 | LINF_320036700 |  | 8 | LINF_360037500 |
| 20 | LINF_360046500 |  | 42 | LINF_320013000 |  | 39 | LINF_300012900 |  | 7 | LINF_050010200 |
| 4 | LINF_180010100 |  | 17 | LINF_090016000 |  | 38 | LINF_300015300 |  | 13 | LINF_360081500 |
| 23 | LINF_330034600 |  | 78 | LINF_060011300 |  | 42 | LINF_330041100 |  | 1 | LINF_330009000 |
| 83 | LINF_360022900 |  | 56 | LINF_350007500 |  | 9 | LINF_290032300 |  | 11 | LINF_150019000 |
| 78 | LINF_060011300 |  | 43 | LINF_350038800 |  | 33 | LINF_200018300 |  | 5 | LINF_140018000 |
| 68 | LINF_060005000 |  | 99 | LINF_320027900 |  | 29 | LINF_100007300 |  | 10 | LINF_350037700 |
| 52 | LINF_340014400 |  | 68 | LINF_060005000 |  | 22 | LINF_230005400 |  | 43 | LINF_270006000 |
| 90 | LINF_350019000 |  | 74 | LINF_360007700 |  | 15 | LINF_360040400 |  | 39 | LINF_300012900 |
| 38 | LINF_060006500 |  | 72 | LINF_200016900 |  | 21 | LINF_270025600 |  | 21 | LINF_270025600 |
| 30 | LINF_350018700 |  | 44 | LINF_190006800 |  | 8 | LINF_360037500 |  | 14 | LINF_350023900 |
| 91 | LINF_190015700 |  | 37 | LINF_150018200 |  | 31 | LINF_170012500 |  | 6 | LINF_320036700 |
| 28 | LINF_270006000 |  | 60 | LINF_100011800 |  | 14 | LINF_350023900 |  | 36 | LINF_070005600 |
| 2 | LINF_330009000 |  | 88 | LINF_350050100 |  | 10 | LINF_350037700 |  | 27 | LINF_330041600 |
| 34 | LINF_190005300 |  | 86 | LINF_300042300 |  | 7 | LINF_050010200 |  | 38 | LINF_300015300 |
| 22 | LINF_270020500 |  | 93 | LINF_140012300 |  | 1 | LINF_330009000 |  | 26 | LINF_130021300 |
| 74 | LINF_360007700 |  | 92 | LINF_210027400 |  | 11 | LINF_150019000 |  | 42 | LINF_330041100 |
| 67 | LINF_350019200 |  | 102 | LINF_360070100 |  | 5 | LINF_140018000 |  | 29 | LINF_100007300 |
| 60 | LINF_100011800 |  | 87 | LINF_360016500 |  | 43 | LINF_270006000 |  | 31 | LINF_170012500 |
| 70 | LINF_260006700 |  | 96 | LINF_340052200 |  | 27 | LINF_330041600 |  | 15 | LINF_360040400 |
| 37 | LINF_150018200 |  | 85 | LINF_070008800 |  | 18 | LINF_190005400 |  | 9 | LINF_290032300 |
| 98 | LINF_300037100 |  | 70 | LINF_260006700 |  | 23 | LINF_210013000 |  | 2 | LINF_170007200 |
| 71 | LINF_040009700 |  | 100 | LINF_210014900 |  | 2 | LINF_170007200 |  | 23 | LINF_210013000 |
| 44 | LINF_190006800 |  | 41 | LINF_330033000 |  | 19 | LINF_360015700 |  | 19 | LINF_360015700 |
| 40 | LINF_180012200 |  | 24 | LINF_100008300 |  | 25 | LINF_060005000 |  | 25 | LINF_060005000 |
| 3 | LINF_320009100 |  | 83 | LINF_360022900 |  | 36 | LINF_070005600 |  | 24 | LINF_350019200 |
| 56 | LINF_350007500 |  | 104 | LINF_300016700 |  | 24 | LINF_350019200 |  | 18 | LINF_190005400 |
| 36 | LINF_070012400 |  | 53 | LINF_210013000 |  | 32 | LINF_300009700 |  | 32 | LINF_300009700 |
| 10 | LINF_310029700 |  | 31 | LINF_190005400 |  |  |  |  |  |  |
| 77 | LINF_140006700 |  | 97 | LINF_070005600 |  |  |  |  |  |  |
| 1 | LINF_170007200 |  | 94 | LINF_29003610 |  |  |  |  |  |  |
| 32 | LINF_210011000 |  | 89 | LINF_290021400 |  |  |  |  |  |  |
| 8 | LINF_270024900 |  | 47 | LINF_280015700 |  |  |  |  |  |  |
| 27 | LINF_100007300 |  | 32 | LINF_210011000 |  |  |  |  |  |  |
| 51 | LINF_220021800 |  | 39 | LINF_110015600 |  |  |  |  |  |  |
| 16 | LINF_340031800 |  | 51 | LINF_220021800 |  |  |  |  |  |  |
| 73 | LINF_110013400 |  | 34 | LINF_190005300 |  |  |  |  |  |  |
| 101 | LINF_330018200 |  | 81 | LINF_230014600 |  |  |  |  |  |  |
| 104 | LINF_300016700 |  | 57 | LINF_240026900 |  |  |  |  |  |  |
| 39 | LINF_110015600 |  | 3 | LINF_320009100 |  |  |  |  |  |  |
| 9 | LINF_300035000 |  | 4 | LINF_180010100 |  |  |  |  |  |  |
| 48 | LINF_220009800 |  | 64 | LINF_020005100 |  |  |  |  |  |  |
| 19 | LINF_360037500 |  | 14 | LINF_340035600 |  |  |  |  |  |  |
| 7 | LINF_150018300 |  | 61 | LINF_240026800 |  |  |  |  |  |  |
| 57 | LINF_240026900 |  | 54 | LINF_160009700 |  |  |  |  |  |  |
| 13 | LINF_050010200 |  | 105 | LINF_360063600 |  |  |  |  |  |  |
| 64 | LINF_020005100 |  | 30 | LINF_350018700 |  |  |  |  |  |  |
| 111 | LINF_350016500 |  | 36 | LINF_070012400 |  |  |  |  |  |  |
| 110 | LINF_360045000 |  | 19 | LINF_360037500 |  |  |  |  |  |  |
| 61 | LINF_240026800 |  | 2 | LINF_330009000 |  |  |  |  |  |  |
| 66 | LINF_160011400 |  | 101 | LINF_330018200 |  |  |  |  |  |  |
| 65 | LINF_360060500 |  | 10 | LINF_310029700 |  |  |  |  |  |  |
| 96 | LINF_340052200 |  | 91 | LINF_190015700 |  |  |  |  |  |  |
| 80 | LINF_360031300 |  | 49 | LINF_280032800 |  |  |  |  |  |  |
| 87 | LINF_360016500 |  | 28 | LINF_270006000 |  |  |  |  |  |  |
| 47 | LINF_280015700 |  | 50 | LINF_320014400 |  |  |  |  |  |  |
| 15 | LINF_290032300 |  | 6 | LINF_290016800 |  |  |  |  |  |  |
| 6 | LINF_290016800 |  | 38 | LINF_060006500 |  |  |  |  |  |  |
| 72 | LINF_200016900 |  | 76 | LINF_180019400 |  |  |  |  |  |  |
| 42 | LINF_320013000 |  | 7 | LINF_150018300 |  |  |  |  |  |  |
| 46 | LINF_300009700 |  | 21 | LINF_350025100 |  |  |  |  |  |  |
| 41 | LINF_330033000 |  | 22 | LINF_270020500 |  |  |  |  |  |  |
| 109 | LINF_350025300 |  | 13 | LINF_050010200 |  |  |  |  |  |  |
| 63 | LINF_240028300 |  | 8 | LINF_270024900 |  |  |  |  |  |  |
| 85 | LINF_070008800 |  | 69 | LINF_260011600 |  |  |  |  |  |  |
| 33 | LINF_350012900 |  | 109 | LINF_350025300 |  |  |  |  |  |  |
| 106 | LINF_330035900 |  | 9 | LINF_300035000 |  |  |  |  |  |  |
| 55 | LINF_320033900 |  | 98 | LINF_300037100 |  |  |  |  |  |  |
| 53 | LINF_210013000 |  | 1 | LINF_170007200 |  |  |  |  |  |  |
| 45 | LINF_130010500 |  | 95 | LINF_110017700 |  |  |  |  |  |  |
| 31 | LINF_190005400 |  | 65 | LINF_360060500 |  |  |  |  |  |  |
| 50 | LINF_320014400 |  | 63 | LINF_240028300 |  |  |  |  |  |  |
| 95 | LINF_110017700 |  | 45 | LINF_130010500 |  |  |  |  |  |  |
| 26 | LINF_270025600 |  | 77 | LINF_140006700 |  |  |  |  |  |  |
| 21 | LINF_350025100 |  | 66 | LINF_160011400 |  |  |  |  |  |  |
| 81 | LINF_230014600 |  | 62 | LINF_130016000 |  |  |  |  |  |  |
| 76 | LINF_180019400 |  | 73 | LINF_110013400 |  |  |  |  |  |  |
| 62 | LINF_130016000 |  | 55 | LINF_320033900 |  |  |  |  |  |  |
| 69 | LINF_260011600 |  | 40 | LINF_180012200 |  |  |  |  |  |  |
| 49 | LINF_280032800 |  | 106 | LINF_330035900 |  |  |  |  |  |  |
| 100 | LINF_210014900 |  | 26 | LINF_270025600 |  |  |  |  |  |  |
| 88 | LINF_350050100 |  | 111 | LINF_350016500 |  |  |  |  |  |  |
| 107 | LINF_130020000 |  | 110 | LINF_360045000 |  |  |  |  |  |  |
| 92 | LINF_210027400 |  | 33 | LINF_350012900 |  |  |  |  |  |  |
| 97 | LINF_070005600 |  | 15 | LINF_290032300 |  |  |  |  |  |  |
| 24 | LINF_100008300 |  | 52 | LINF_340014400 |  |  |  |  |  |  |
| 102 | LINF_360070100 |  | 48 | LINF_220009800 |  |  |  |  |  |  |
| 93 | LINF_140012300 |  | 107 | LINF_130020000 |  |  |  |  |  |  |
| 86 | LINF_300042300 |  | 16 | LINF_340031800 |  |  |  |  |  |  |
| 89 | LINF_290021400 |  | 67 | LINF_350019200 |  |  |  |  |  |  |
| 58 | LINF_230005400 |  | 84 | LINF_180020700 |  |  |  |  |  |  |
| 35 | LINF_360015100 |  | 20 | LINF_360046500 |  |  |  |  |  |  |
| 103 | LINF_350052900 |  | 18 | LINF_150018600 |  |  |  |  |  |  |
| 84 | LINF_180020700 |  | 11 | LINF_350005300 |  |  |  |  |  |  |
| 25 | LINF_360015700 |  | 5 | LINF_360048000 |  |  |  |  |  |  |
| 94 | LINF_29003610 |  | 80 | LINF_360031300 |  |  |  |  |  |  |

**Supplementary Table 2.** Gene ID of the proteins identified in *L.infantum* WT strain (82 proteins) and MF-resistant strains (51 proteins). The order of the proteins in each condition represents the protein's order that appears in the heat map for miltefosine.

| WT | |  | WT+MF | |  | MF200.5 | |  | MF200.5+MF | |
| --- | --- | --- | --- | --- | --- | --- | --- | --- | --- | --- |
| Order | Gene ID |  | Order | Gene ID |  | Order | Gene ID |  | Order | Gene ID |
| 65 | LINF_360022900 |  | 46 | LINF_360066000 |  | 42 | LINF_200018300 |  | 46 | LINF_350023200 |
| 5 | LINF_100010800 |  | 8 | LINF_360048000 |  | 7 | LINF_150019000 |  | 23 | LINF_270032200 |
| 8 | LINF_360048000 |  | 64 | LINF_360016500 |  | 35 | LINF_300042300 |  | 47 | LINF_100015600 |
| 49 | LINF_210027500 |  | 60 | LINF_110013400 |  | 23 | LINF_270032200 |  | 26 | LINF_360069000 |
| 55 | LINF_320040100 |  | 3 | LINF_170007200 |  | 48 | LINF_140015400 |  | 15 | LINF_320014400 |
| 53 | LINF_060016300 |  | 69 | LINF_160006500 |  | 10 | LINF_250014900 |  | 17 | LINF_160013000 |
| 4 | LINF_250018000 |  | 30 | LINF_060006500 |  | 47 | LINF_100015600 |  | 49 | LINF_330018200 |
| 25 | LINF_330034600 |  | 12 | LINF_210007900 |  | 33 | LINF_360079400 |  | 44 | LINF_140012300 |
| 32 | LINF_110015600 |  | 65 | LINF_360022900 |  | 39 | LINF_270027100 |  | 37 | LINF_330013000 |
| 12 | LINF_210007900 |  | 5 | LINF_100010800 |  | 30 | LINF_300041800 |  | 48 | LINF_140015400 |
| 37 | LINF_350021700 |  | 55 | LINF_320040100 |  | 3 | LINF_360048000 |  | 2 | LINF_170007200 |
| 59 | LINF_320007400 |  | 74 | LINF_290027200 |  | 28 | LINF_090016900 |  | 1 | LINF_330009000 |
| 2 | LINF_330009000 |  | 66 | LINF_200021000 |  | 17 | LINF_160013000 |  | 6 | LINF_300035000 |
| 33 | LINF_360015700 |  | 48 | LINF_180019400 |  | 8 | LINF_260011400 |  | 27 | LINF_020005100 |
| 9 | LINF_360018400 |  | 25 | LINF_330034600 |  | 13 | LINF_320013000 |  | 34 | LINF_260006700 |
| 36 | LINF_180012200 |  | 33 | LINF_360015700 |  | 1 | LINF_330009000 |  | 18 | LINF_110015600 |
| 24 | LINF_120010600 |  | 6 | LINF_320009100 |  | 9 | LINF_340031800 |  | 36 | LINF_060011300 |
| 78 | LINF_120016900 |  | 14 | LINF_090016000 |  | 11 | LINF_070012400 |  | 24 | LINF_300036500 |
| 41 | LINF_100011800 |  | 21 | LINF_130017300 |  | 2 | LINF_170007200 |  | 50 | LINF_320027900 |
| 30 | LINF_060006500 |  | 79 | LINF_340052200 |  | 51 | LINF_070015900 |  | 14 | LINF_360015700 |
| 35 | LINF_330033000 |  | 16 | LINF_310029700 |  | 40 | LINF_050009500 |  | 4 | LINF_280034700 |
| 13 | LINF_030006800 |  | 2 | LINF_330009000 |  | 32 | LINF_240021800 |  | 45 | LINF_070005600 |
| 63 | LINF_200018300 |  | 18 | LINF_260017400 |  | 22 | LINF_150008100 |  | 33 | LINF_360079400 |
| 74 | LINF_290027200 |  | 45 | LINF_240026900 |  | 36 | LINF_060011300 |  | 5 | LINF_290016800 |
| 34 | LINF_320013000 |  | 4 | LINF_250018000 |  | 25 | LINF_240005200 |  | 22 | LINF_150008100 |
| 27 | LINF_350018700 |  | 49 | LINF_210027500 |  | 38 | LINF_290036400 |  | 20 | LINF_350042400 |
| 16 | LINF_310029700 |  | 15 | LINF_340031800 |  | 24 | LINF_300036500 |  | 16 | LINF_150018200 |
| 7 | LINF_180010100 |  | 9 | LINF_360018400 |  | 34 | LINF_260006700 |  | 38 | LINF_290036400 |
| 52 | LINF_260011600 |  | 71 | LINF_180007100 |  | 6 | LINF_300035000 |  | 31 | LINF_040009700 |
| 50 | LINF_060006300 |  | 22 | LINF_040012600 |  | 14 | LINF_360015700 |  | 21 | LINF_160009700 |
| 39 | LINF_100017000 |  | 7 | LINF_180010100 |  | 4 | LINF_280034700 |  | 40 | LINF_050009500 |
| 38 | LINF_190005400 |  | 37 | LINF_350021700 |  | 5 | LINF_290016800 |  | 12 | LINF_080015800 |
| 61 | LINF_310036600 |  | 17 | LINF_350025100 |  | 41 | LINF_110017900 |  | 28 | LINF_090016900 |
| 80 | LINF_330035900 |  | 63 | LINF_200018300 |  | 27 | LINF_020005100 |  | 11 | LINF_070012400 |
| 17 | LINF_350025100 |  | 62 | LINF_360047300 |  | 21 | LINF_160009700 |  | 3 | LINF_360048000 |
| 56 | LINF_130021300 |  | 39 | LINF_100017000 |  | 19 | LINF_230005400 |  | 35 | LINF_300042300 |
| 20 | LINF_010009300 |  | 34 | LINF_320013000 |  | 50 | LINF_320027900 |  | 42 | LINF_200018300 |
| 22 | LINF_040012600 |  | 78 | LINF_120016900 |  | 45 | LINF_070005600 |  | 25 | LINF_240005200 |
| 19 | LINF_350047100 |  | 54 | LINF_240012700 |  | 18 | LINF_110015600 |  | 41 | LINF_110017900 |
| 40 | LINF_040009700 |  | 53 | LINF_060016300 |  | 31 | LINF_040009700 |  | 32 | LINF_240021800 |
| 72 | LINF_140012300 |  | 67 | LINF_350019000 |  | 20 | LINF_350042400 |  | 51 | LINF_070015900 |
| 11 | LINF_280034700 |  | 43 | LINF_050009500 |  | 15 | LINF_320014400 |  | 13 | LINF_320013000 |
| 77 | LINF_290031100 |  | 76 | LINF_190006300 |  | 16 | LINF_150018200 |  | 9 | LINF_340031800 |
| 68 | LINF_290021400 |  | 38 | LINF_190005400 |  | 44 | LINF_140012300 |  | 19 | LINF_230005400 |
| 48 | LINF_180019400 |  | 28 | LINF_360015100 |  | 43 | LINF_210027400 |  | 8 | LINF_260011400 |
| 14 | LINF_090016000 |  | 57 | LINF_060011300 |  | 37 | LINF_330013000 |  | 10 | LINF_250014900 |
| 1 | LINF_170007200 |  | 81 | LINF_330041000 |  | 49 | LINF_330018200 |  | 43 | LINF_210027400 |
| 23 | LINF_350027200 |  | 31 | LINF_220009800 |  | 12 | LINF_080015800 |  | 39 | LINF_270027100 |
| 66 | LINF_200021000 |  | 1 | LINF_170007200 |  | 26 | LINF_360069000 |  | 30 | LINF_300041800 |
| 75 | LINF_070005600 |  | 40 | LINF_040009700 |  | 46 | LINF_350023200 |  | 7 | LINF_150019000 |
| 57 | LINF_060011300 |  | 75 | LINF_070005600 |  | 29 | LINF_320015100 |  | 29 | LINF_320015100 |
| 73 | LINF_130013700 |  | 32 | LINF_110015600 |  |  |  |  |  |  |
| 69 | LINF_160006500 |  | 20 | LINF_010009300 |  |  |  |  |  |  |
| 29 | LINF_080015800 |  | 23 | LINF_350027200 |  |  |  |  |  |  |
| 76 | LINF_190006300 |  | 82 | LINF_330026200 |  |  |  |  |  |  |
| 47 | LINF_350042400 |  | 61 | LINF_310036600 |  |  |  |  |  |  |
| 26 | LINF_110016000 |  | 10 | LINF_300035000 |  |  |  |  |  |  |
| 81 | LINF_330041000 |  | 26 | LINF_110016000 |  |  |  |  |  |  |
| 79 | LINF_340052200 |  | 59 | LINF_320007400 |  |  |  |  |  |  |
| 45 | LINF_240026900 |  | 80 | LINF_330035900 |  |  |  |  |  |  |
| 70 | LINF_350043800 |  | 70 | LINF_350043800 |  |  |  |  |  |  |
| 67 | LINF_350019000 |  | 19 | LINF_350047100 |  |  |  |  |  |  |
| 64 | LINF_360016500 |  | 13 | LINF_030006800 |  |  |  |  |  |  |
| 28 | LINF_360015100 |  | 11 | LINF_280034700 |  |  |  |  |  |  |
| 10 | LINF_300035000 |  | 77 | LINF_290031100 |  |  |  |  |  |  |
| 21 | LINF_130017300 |  | 24 | LINF_120010600 |  |  |  |  |  |  |
| 6 | LINF_320009100 |  | 52 | LINF_260011600 |  |  |  |  |  |  |
| 71 | LINF_180007100 |  | 56 | LINF_130021300 |  |  |  |  |  |  |
| 44 | LINF_330013000 |  | 51 | LINF_130016000 |  |  |  |  |  |  |
| 15 | LINF_340031800 |  | 27 | LINF_350018700 |  |  |  |  |  |  |
| 43 | LINF_050009500 |  | 47 | LINF_350042400 |  |  |  |  |  |  |
| 31 | LINF_220009800 |  | 58 | LINF_340005600 |  |  |  |  |  |  |
| 62 | LINF_360047300 |  | 41 | LINF_100011800 |  |  |  |  |  |  |
| 51 | LINF_130016000 |  | 36 | LINF_180012200 |  |  |  |  |  |  |
| 60 | LINF_110013400 |  | 68 | LINF_290021400 |  |  |  |  |  |  |
| 46 | LINF_360066000 |  | 44 | LINF_330013000 |  |  |  |  |  |  |
| 18 | LINF_260017400 |  | 73 | LINF_130013700 |  |  |  |  |  |  |
| 58 | LINF_340005600 |  | 72 | LINF_140012300 |  |  |  |  |  |  |
| 82 | LINF_330026200 |  | 50 | LINF_060006300 |  |  |  |  |  |  |
| 54 | LINF_240012700 |  | 35 | LINF_330033000 |  |  |  |  |  |  |
| 42 | LINF_030014900 |  | 29 | LINF_080015800 |  |  |  |  |  |  |
| 3 | LINF_170007200 |  | 42 | LINF_030014900 |  |  |  |  |  |  |

**Supplementary Table 3.** Gene ID of the proteins identified in *L.infantum* WT strain (14 proteins) and AmB-resistant strains (40 proteins). The order of the proteins in each condition represents the protein's order that appears in the heat map for amphotericin B.

| WT | |  | WT+AmB | |  | AmB1000.1 | |  | AmB1000.1+AmB | |
| --- | --- | --- | --- | --- | --- | --- | --- | --- | --- | --- |
| Order | Gene ID |  | Order | Gene ID |  | Order | Gene ID |  | Order | Gene ID |
| 6 | LINF_360050900 |  | 11 | LINF_120005200 |  | 39 | LINF_270006800 |  | 39 | LINF_270006800 |
| 3 | LINF_360048000 |  | 3 | LINF_360048000 |  | 23 | LINF_330034600 |  | 19 | LINF_210013000 |
| 12 | LINF_290015200 |  | 6 | LINF_360050900 |  | 38 | LINF_100015200 |  | 23 | LINF_330034600 |
| 9 | LINF_300036900 |  | 10 | LINF_200021000 |  | 15 | LINF_040012600 |  | 13 | LINF_100008300 |
| 14 | LINF_170016800 |  | 7 | LINF_350020400 |  | 8 | LINF_050010200 |  | 38 | LINF_100015200 |
| 10 | LINF_200021000 |  | 2 | LINF_330009000 |  | 32 | LINF_200006000 |  | 30 | LINF_230005400 |
| 2 | LINF_330009000 |  | 4 | LINF_230005400 |  | 10 | LINF_340031800 |  | 37 | LINF_350019000 |
| 1 | LINF_170007200 |  | 12 | LINF_290015200 |  | 11 | LINF_170005000 |  | 26 | LINF_220021800 |
| 8 | LINF_130021300 |  | 8 | LINF_130021300 |  | 2 | LINF_360007100 |  | 14 | LINF_350025100 |
| 5 | LINF_220009800 |  | 5 | LINF_220009800 |  | 36 | LINF_300042300 |  | 32 | LINF_200006000 |
| 13 | LINF_270012500 |  | 1 | LINF_170007200 |  | 17 | LINF_350043300 |  | 1 | LINF_330009000 |
| 7 | LINF_350020400 |  | 13 | LINF_270012500 |  | 26 | LINF_220021800 |  | 10 | LINF_340031800 |
| 4 | LINF_230005400 |  | 9 | LINF_300036900 |  | 18 | LINF_260013800 |  | 5 | LINF_300035000 |
| 11 | LINF_120005200 |  | 14 | LINF_170016800 |  | 12 | LINF_130017300 |  | 36 | LINF_300042300 |
|  |  |  |  |  |  | 5 | LINF_300035000 |  | 8 | LINF_050010200 |
|  |  |  |  |  |  | 14 | LINF_350025100 |  | 24 | LINF_350018700 |
|  |  |  |  |  |  | 3 | LINF_170007200 |  | 17 | LINF_350043300 |
|  |  |  |  |  |  | 1 | LINF_330009000 |  | 12 | LINF_130017300 |
|  |  |  |  |  |  | 33 | LINF_110017200 |  | 3 | LINF_170007200 |
|  |  |  |  |  |  | 20 | LINF_320040200 |  | 27 | LINF_320033900 |
|  |  |  |  |  |  | 21 | LINF_070012400 |  | 11 | LINF_170005000 |
|  |  |  |  |  |  | 29 | LINF_110015600 |  | 29 | LINF_110015600 |
|  |  |  |  |  |  | 27 | LINF_320033900 |  | 18 | LINF_260013800 |
|  |  |  |  |  |  | 24 | LINF_350018700 |  | 2 | LINF_360007100 |
|  |  |  |  |  |  | 6 | LINF_280034700 |  | 16 | LINF_150017600 |
|  |  |  |  |  |  | 28 | LINF_120010600 |  | 9 | LINF_270024900 |
|  |  |  |  |  |  | 7 | LINF_340014000 |  | 6 | LINF_280034700 |
|  |  |  |  |  |  | 34 | LINF_320015700 |  | 25 | LINF_320013000 |
|  |  |  |  |  |  | 22 | LINF_060009600 |  | 28 | LINF_120010600 |
|  |  |  |  |  |  | 9 | LINF_270024900 |  | 40 | LINF_060013800 |
|  |  |  |  |  |  | 16 | LINF_150017600 |  | 21 | LINF_070012400 |
|  |  |  |  |  |  | 4 | LINF_360020500 |  | 7 | LINF_340014000 |
|  |  |  |  |  |  | 40 | LINF_060013800 |  | 15 | LINF_040012600 |
|  |  |  |  |  |  | 25 | LINF_320013000 |  | 31 | LINF_110016300 |
|  |  |  |  |  |  | 31 | LINF_110016300 |  | 22 | LINF_060009600 |
|  |  |  |  |  |  | 30 | LINF_230005400 |  | 4 | LINF_360020500 |
|  |  |  |  |  |  | 37 | LINF_350019000 |  | 20 | LINF_320040200 |
|  |  |  |  |  |  | 13 | LINF_100008300 |  | 34 | LINF_320015700 |
|  |  |  |  |  |  | 35 | LINF_350014400 |  | 33 | LINF_110017200 |
|  |  |  |  |  |  | 19 | LINF_210013000 |  | 35 | LINF_350014400 |
